# Generic medicines: an evaluation of the accuracy and accessibility of information available on the internet

**DOI:** 10.1186/1472-6947-13-115

**Published:** 2013-10-07

**Authors:** Suzanne S Dunne, Niamh M Cummins, Ailish Hannigan, Bill Shannon, Colum Dunne, Walter Cullen

**Affiliations:** 1 Graduate Entry Medical School, University of Limerick, Limerick, Ireland; 2 Centre for Interventions in Infection, Inflammation and Immunity (4i), Graduate Entry Medical School, University of Limerick, Limerick, Ireland

**Keywords:** Generic medicine, Internet, Medical information, Patient education, Google, Readability

## Abstract

**Background:**

Internationally, generic medicines are increasingly seen as a key strategy to reduce healthcare expenditure, therefore awareness and knowledge transfer regarding generic medicines are valid areas of research. Although the Internet is a frequently used source of medical information, the accuracy of material found online is variable. The aim of this study was to evaluate information provided on the Internet regarding generic medicines in terms of quality of information and readability.

**Methods:**

Internet searches for information regarding generic medicine were completed, with a pre-defined search term, using the Google search engine, in five English-speaking geographical regions (US, UK, Ireland, Canada and Australia). Search results likely to be looked at by a searcher were collated and assessed for the quality of generic medicine-related information in the websites, using a novel customised Website Quality Assessment (WQA) tool; and for readability, using existing methods. The reproducibility of the tools between two independent reviewers was evaluated and correlations between WQA score, readability statistics and Google search engine results page ranking were assessed.

**Results:**

Wikipedia was the highest-ranking search result in 100% of searches performed. Considerable variability of search results returned between different geographical regions was observed, including that websites identified in the Australian search generated the highest number of country specific websites; searches performed using computers with Irish, British, American and Canadian IP addresses appear to be more similar to each other than the google.com search performed in Australia; and the Canadian google.ca results show a notable difference from any of the other searches. Of the 24 websites assessed, none scored a perfect WQA score. Notably, strong correlation was seen between WQA and readability scores and ranking on google.com search results.

**Conclusions:**

This novel evaluation of websites providing information on generic medicines showed that, of the websites likely to be seen by a searcher, none demonstrated a combination of scoring highly on quality of information (as evinced by WQA score) *and* readability. Therefore, there is a gap in online knowledge provision on this topic which, if filled by a website designed using the WQA tool developed in this study, has an improved likelihood of ranking highly in google.com search results.

## Background

The Internet has become a source of medical information for patients and healthcare professionals alike. However, the accuracy of information found online may not always necessarily be relied on, and concerns have been raised about the quality of information that may be found, by patients, on the Internet [1-3]. With healthcare costs soaring, many governments are increasingly making use of generic medicines to constrain expenditure. Additionally, many commonly used proprietary medicines have recently, or will in the near future, hit the so-called “patent cliff” – thus enabling generic competition [4-6]. As a result, patients are increasingly likely to be prescribed generic medicines, possibly in place of more familiar proprietary brands. When a patient has medical or healthcare queries, such as questions about generic medicines, the Internet is likely to be one of the first places they will seek information [7]. Therefore, the question should be asked: Is information available on the Internet regarding generic medicines accurate, accessible and of good quality? (For the purposes of this study, the term “accessibility” is used in the context of how readable and understandable the information provided is to the lay reader).

Studies have assessed the use, quality and/or availability of medical/healthcare information available on the Internet in areas as diverse as: inflammatory bowel disease [8], orthodontistry, [9,10] pain, [11] cancer, [12-14] and mental health, [15,16] amongst many others. Such studies often limit themselves to assessing available information in particular countries [17-20]. As many misconceptions exist about generic medicines, and given that healthcare professionals have expressed poor opinions of generics in the past, [21] there is a challenge in ensuring that accurate and relevant information is communicated to the general public. This challenge includes not only the necessity to provide accurate information, to dispel myths and to counter misinformation, but also to present material in a manner that is accessible to the intended audience. It has been reported that, in the case of patients particularly, myths and questions remain about generic medicines, and that accurate information can be difficult to source [22].

The aim of this study was to evaluate the availability of information on the Internet regarding generic medicines. This study additionally assessed whether the information in websites likely to be looked at by patients is accurate (as measured by use of a website quality assessment (WQA) tool) and accessible (as determined by readability and understandability statistics). While much information exists on research into the provision of medical information on the Internet, to the authors’ knowledge, no evaluation has been published specifically regarding quality and accessibility of information on generic medicines. This study aimed to bridge that knowledge gap while evaluating availablity and accessibility of that information in several English-speaking countries.

## Methods

### Choice of search engine

StatCounter, a web analytics company, reported in their “GlobalStats graph” that - for the 12 month period from Jan 2012 to Jan 2013 - Google was the most commonly used search engine globally, holding approximately 90% of the worldwide search engine market [23]. Therefore, as Google is the search engine of choice globally, Google was the search engine used for this study.

### Choice of search term

A patient wishing to make an Internet enquiry about a generic medicine is likely to use either the term “generic drug”, or “generic medicine” as their search term. To accommodate both of these, the search term used for this study was “generic medicine OR drug” (without the quotation marks, and with the “OR” capitalised).

The reasoning for this is that Google’s default behaviour is to consider all the words in a search. In order to allow *either* of the words “drug” or “medicine” to be searched for, the “OR” operator can be used (the OR must be in CAPS). With this search, Google will return SERP (search engine results page) hits that contain the word “generic” and either of the words “drug” or “medicine”. Without the “OR” operator, Google would only return pages that have both the words “drug” and “medicine” on the page, as the “AND” operator is the default [24].

All searches were performed during March and April of 2012.

### Inclusion of web sites

A study from 2008 showed that 68% of search engine users click a search result within the first page of results (the default for Google is 10 results per page), and are unlikely to go to the second page of results [25,26]. Therefore, following a search using the defined search term, the results on the first SERP returned that met the following inclusion criteria were assessed: (i) web site written in English; (ii) web site not being a portal providing links to third party sites; (iii) web site not a news story (e.g., as found by Google news search); (iv) web site not a sales website and (v) website not spurious and being related to the topic of generic medicines.

### Determination of global variability

To assess global variability, searches were performed in regional Google search engines, google.ie (Ireland), google.co.uk (United Kingdom), google.ca (Canada), and google.com.au (Australia) in additional to the main US site: google.com.

To assess if Internet Protocol (IP) addresses have any impact on the results obtained, searches were also performed using google.com, on computers in the following five regions: Ireland, United States, Great Britain, Australia, and Canada.

IP stands for “Internet Protocol”. Every device (e.g., computer, tablet, printer etc.) on a computer network has a unique, numeric identifier called an IP address. Similarly to how someone sending a letter would write the intended address on the envelope, a computer’s IP address is used to identify and locate that specific device on a computer network, or on the Internet [27].

Overall, two searches (that is: on the local and .com sites) were performed on computers with an IP address in each of the five regions above, meaning that a total of nine searches were completed.

### Assessment of quality of information

The questions in Table 1 – Website Quality Assessment (WQA) Questions for Website Information were asked in relation to each website. The WQA tool was developed for this study as, to the authors’ knowledge, no previous assessment of websites providing information about generic medicines had been published. The WQA tool consists of 22 yes/no type questions, with a point awarded for positive or correct information. No points are awarded where information was lacking, or for inaccurate information. Questions that could not be answered were designated “not applicable” (N/A) and no score awarded. An overall WQA score for each website was totalled from the scores given to each assessment question. (In some cases, just the initial page linked to in the Google search was assessed, however, in the cases where clear and relevant links to other pages containing information of interest within the same website were obvious to the searcher, these were also assessed).

**Table 1:** Website quality assessment (WQA) questions for assessing generic medicine website information

**Question**	**Answer and score**	**WQA score awarded**
Does the site give an explanation as to what a generic medicine is?	Yes = 1	
	No = 0	
Is this explanation correct? (i.e. equivalent in dose, strength, route of administration, safety, efficacy, and intended use)	Yes = 1	
	No = 0	
If so, is the explanation of a generic medicine readable and understandable by a non-scientist?	Yes = 1	
	No = 0	
Are examples given of generic medicines? E.g. example of a proprietary medicine that also state their counterpart generic medicine?	Yes = 1	
	No = 0	
Is bioequivalence mentioned in the website?	Yes = 1	
	No = 0	
Is bioequivalence explained?	Yes = 1	
	No = 0	
	N/A	
If so, is the explanation of bioequivalence correct?	Yes = 1	
	No = 0	
	N/A	
If so, is the explanation of bioequivalence readable and understandable by a non-scientist?	Yes = 1	
	No = 0	
	N/A	
Is the cheaper price of generics referred to?	Yes = 1	
	No = 0	
Is an *accurate* reason for the cheaper price of generics given?	Yes = 1	
	No = 0	
	N/A	
Is any *inaccurate* information regarding the cheaper price of generics given?	Yes = 0	
	No = 1	
	N/A	
Are examples given of the actual price difference between generics and proprietary medicines, or of the amount of money that can be saved by use of generics?	Yes = 1	
	No = 0	
Is reference made to the fact that approved, equivalent generic meds can have a different appearance (colour, shape etc.) different taste/smell or different inactive ingredients?	Yes = 1	
	No = 0	
Are narrow therapeutic index [18] drugs mentioned?	Yes = 1	
	No = 0	
Is the difference between NTI and non-NTI drugs explained?	Yes = 1	
	No = 0	
	N/A	
Is there *accurate* information given on how generic bioequivalence, or generic manufacturing may affect NTI drugs?	Yes = 1	
	No = 0	
	N/A	
Is any *inaccurate* information given regarding NTI drugs?	Yes = 0	
	No = 1	
	N/A	
Are “pros” of generics mentioned? [e.g. lower price for same safety & bioequivalence etc.…]	Yes = 1	
	No = 0	
Are any “cons” of generics mentioned? [e.g. adverse events to dissimilar excipients etc.…]	Yes = 1	
	No = 0	
Is the difference between proprietary and non-proprietary names mentioned?	Yes = 1	
	No = 0	
Is the explanation given for the difference between proprietary and non-proprietary names accurate?	Yes = 1	
	No = 0	
	N/A	
Generic prescribing mentioned and explained accurately?	Yes = 1	
	No = 0	
Total score		
Flesch reading ease score		
Flesch Kinkaid grade level		

The WQA questions were designed to account for all of the information that a patient might need in order to accurately answer any questions they may have about generic drugs, for example: an explanation as to what a generic drug is and how it differs from a proprietary drug – including price, appearance etc.; explanation of bioequivalence; examples of generic drugs and their proprietary counterparts; information regarding when generic substitution may not be appropriate – e.g., in the case of narrow therapeutic index drugs and any pros or cons of generic medicines.

### Assessment of website accessibility

A minimum of a 100-word sample of continuous text from each of the websites was extracted and pasted into Microsoft Word. This text was then analysed using the Flesch Reading Ease score [28] in the MS Word application.

MS Word’s Flesch Reading Ease score is based on a formula developed in 1948 by Rudolf Flesch and determines readibility [28]. It is computed using the average number of syllables per word and words per sentence. Syllables-per-word is a measure of word difficulty. Words-per-sentence is an indicator of syntactic complexity.

The Flesch Reading Ease scale ranges from zero to 100. Zero to 50 is very difficult to difficult reading. Eighty and above is easy to very easy reading. Flesch set the minimum score for plain English at 60 [28]. Microsoft’s documentation encourages authors of standard documents to aim for a score of 60 to 70 [29,30].

Additionally, the Flesch-Kincaid Grade Level was used to determine the understandability of each website. The Flesch-Kincaid Grade Level, which was developed in 1975, measures the readability of a document based on the minimum education level required for a reader to understand it [31]. Microsoft recommends aiming for a Flesch-Kincaid score of 7.0 to 8.0 for most documents. According to a 1993 study, the average adult in the U.S. reads at the seventh- grade level and the authors of that study recommended that materials for the public be written at a fifth- or sixth-grade reading level [29].

### Statistical analyses

Two reviewers rated each selected website independently and their scores were compared to assess reproducibility of the WQA tool and the readability assessments. Using Statistical Packages for the Social Sciences (version 20.0), the intra-class correlation coefficient (ICC) was used to measure reproducibility. Spearman’s correlation coefficient (r_s_) was used to measure the association between the ranking of websites with WQA scores and readability assessments. Absolute values of r_s_ > 0.3 were considered to represent moderate correlations, > 0.5 were considered strong correlations. The scores from the developer of the assessment tool (SD) were used in the correlation analyses. The correlation between ranking of websites and WQA scores was also used to demonstrate the predictive validity of this newly developed assessment tool.

## Results

### Determination of websites for assessment

Thirty-eight (38) unique hits (i.e. individual search results) were identified from the first SERPs of the nine searches performed. Of these, 15 hits were discarded for the reasons described in the methodology or were amalgamated with another hit. (For example, the website entitled: *EGA - Basics of generic medicines* was a hit on both of the IE searches. Additionally, the main EGA website was a hit on the google.co.uk search. As both relate to the same website, the results were combined into one and the EGA website assessed as a single site, rather than individual pages).

An additional website – entitled *Generics Are The Same* - was added during the rating exercise as it was directly referred to in the *Canadian Generic Pharmaceutical Association* website and is also published by the Canadian Generic Pharmaceutical Association. As the *Generics Are The Same* website is the explanatory arm of the *Canadian Generic Pharmaceutical Association* website it was decided to also assess this website as a patient accessing the first website is very likely to follow links through to the second. This was the only example of an associated website being assessed.

Overall, a total of 24 individual websites were assessed using the WQA tool. Results of the assessments for each of the 24 websites are displayed in Table 2, which additionally shows the ranking on the Google search results page for each website assessed, in each of the individual domain searches.

**Table 2:** Websites assessed with their rankings on the different google searches and website quality assessment (WQA) score

	**Website title**	**Google SERP ranking**^**a**^									**WQA score**	**Flesch reading ease score**	**Flesch Kinkaid grade level**
		**IE / .com**	**IE / .ie**	**UK / .com**	**UK / .co.uk**	**US / .com**	**CA / .com**	**CA / .ca**	**AU / .com**	**AU/ .com.au**			
1	Generic drug - Wikipedia, the free encyclopedia	1	1	1	1	1	1	1	1	1	**16**	**49.1**	**10.2**
2	Generic Drugs: Know the Benefits and Differences of Generic Drugs – about.com	2	2	2	3	2	2		10	6	**16**	**53.5**	**8**
3	Generic drugs, Are They as Good as Brand Names? - MedicineNet.com	3	3	3	5	5	3		3	2	**11**	**42.5**	**11.3**
4	Understanding Generic Drugs	4	5	4	6	3	4		6	7	**16**	**57.2**	**9**
5	Branded and generic medicines	5	7	6	2		6			9	**17**	**36.7**	**14.6**
6	WHO | Generic Drugs	6	4	5	7		5		5	5	**10**	**32.9**	**13.7**
7	RxList – Facts About Generic Drugs	7	6			4			8		**9**	**79.3**	**4.6**
8	EGA - European Generic medicines Association	8	9		8						**13**	**22.9**	**12**
9	National Medicines Information Centre - Generic Prescribing		8								**17**	**31**	**11.5**
10	Generic Drugs - What are Generic Drugs?		10								**15**	**25**	**17.1**
11	GPhA - Generic Pharmaceutical Association			9		7	9				**14**	**39.4**	**14.5**
12	Generic / Brand Drug Name Table			10			10	3			**10**	**48.6**	**10**
13	Generic vs Brand Name Medicines				4						**9**	**60**	**8**
14	AIDS, Drug Prices and Generic Drugs				9	10					**11**	**27**	**15**
15	Canadian Generic Pharmaceutical Association							2			**12**	**55.6**	**9.3**
16	Generics Are The Same							(2)^b^			**12**	**43.7**	**12.5**
17	Generic Drugs In Canada: A Policy Paper							4			**8**	**37.3**	**14**
18	Benefiting from Generic Drug Competition in Canada: The Way Forward							7			**11**	**54.4**	**7.7**
19	Generic Drugs – The Same Medicine for Less Money								7		**10**	**75.1**	**5.6**
20	Generic Drugs								9		**6**	**42**	**11.9**
21	The Generic Medicines industry Association of Australia									3	**13**	**24.4**	**12**
22	Australian Prescriber: Frequently asked questions about generic medicines									4	**15**	**22.2**	**12**
23	Pricing of PBS Medicine - Medicare Australia									8	**4**	**34.3**	**12**
24	Questions and answers on generic medicines									10	**10**	**32.9**	**12**

^a^Abbreviations used: *IE* Ireland, *UK* United Kingdom, *US* United States, *CA* Canada, *AU* Australia.

^b^This website “Generics Are The Same” was not a result in the original searches, but was directly linked to the Canadian Generic Pharmaceutical Association website, which was the second result returned in the google.ca search. As it is likely that a patient finding the first website would link into the second, it was added to this study and WQA assessed with the other websites found.

### Analysis of websites from search results

Visual analysis of the search results (Table 2), including comparison of the international searches, showed that Wikipedia (a collaboratively edited, multilingual, free Internet encyclopedia supported by the non-profit Wikimedia Foundation) was the number one ranked search result in all searches completed. This is consistent with findings in other studies [32,33] including a study reporting that Wikipedia is the 6th most accessed website on the Internet globally [34] and, therefore, likely to be visited by those seeking medical information.

After Wikipedia, the following five websites were the most likely to be used by searchers, based on the search terms used in this study:

•About.com’s page entitled *Generic Drugs: Know the Benefits and Differences of Generic Drugs*

•MedicineNet.com’s page: *Generic Drugs, Are They as Good as Brand Names?*

•The US Food and Drug Administration (FDA)’s page entitled *Understanding Generic Drugs*

•NetDoctor.co.uk’s page entitled *Branded and generic medicines*

•The World Health Organisation’s page: *Generic Drugs*

These six websites (Wikipedia and the five others above that appear most often) all appear in at least six of the nine searches completed (Table 2).

SERPs returned to searchers during this study demonstrate that a search, using identical search terms and performed in the local Google search engine, compared to that performed on the same computer (i.e., same IP address) but in the google.com domain, can provide substantially different results (Table 2). Other notable observations from Table 2 include that the European Generic Medicines Association (EGA) website was a hit in three of the four searches conducted in Europe (it was not a result in the google.com UK search) but was not seen in any of the other searches. This could indicate a possible regional variance. However, the FDA website (an American website) was a hit in all searches, with the exception of the Canadian google.ca search (it was a hit in the Canadian google.com search). Additionally, it was noted that the results of the Australian searches had the highest level of country/domain specific websites with six unique hits observed between the Australian google.com and google.com.au searches that were not seen elsewhere.

The google.com searches performed using computers with Irish, British, American and Canadian IP addresses appear to be more similar to each other than the google.com search performed in Australia. The Australian google.com profile is noticeably different from the other search results with two unique websites not present in the other google.com searches.

Interestingly, the Canadian google.ca results show a distinct difference from any of the other searches due to the absence of most of the websites seen in other regional searches, with the exception of Wikipedia and Canadian websites.

### WQA scores

The WQA tool (Table 1) employed 22 yes/no type questions to assess the quality of information contained in each website. From a maximum available score of 22, the highest score awarded was 17 - awarded to two websites: (i) Netdoctor.co.uk – *Branded and generic medicines* and (ii) The Irish National Information Centre’s publication - *Generic Prescribing* (websites numbered 5 and 9 respectively in Table 3). However, only the Netdoctor.co.uk site was also in the top six sites indicated by the Google search rankings. The Irish National Information Centre’s publication was a result only in the google.ie (Irish IP address) search, and its likelihood of being seen outside Ireland considered small*.*

**Table 3:** WQA assessed website titles and associated URLs

**Website number**	**Website title**	**URL**
1	Generic drug - Wikipedia, the free encyclopedia	http://en.wikipedia.org/wiki/Generic_drug
2	Generic Drugs: Know the Benefits and Differences of Generic Drugs	http://patients.about.com/od/drugsandsafety/a/genericdrugs.htm
3	Generic drugs, Are They as Good as Brand Names?	http://www.medicinenet.com/script/main/art.asp?articlekey=46204
4	Understanding Generic Drugs	http://www.fda.gov/Drugs/ResourcesForYou/Consumers/BuyingUsingMedicineSafely/UnderstandingGenericDrugs/default.htm
5	Branded and generic medicines	http://www.netdoctor.co.uk/medicines/brand_generic.htm
6	WHO - Generic Drugs	http://www.who.int/trade/glossary/story034/en/index.html
7	RxList – Facts About Generic Drugs	http://www.rxlist.com/script/main/art.asp?articlekey=81666
8	EGA - European Generic medicines Association	http://www.egagenerics.com
9	National Medicines Information Centre - Generic Prescribing	http://www.stjames.ie/GPsHealthcareProfessionals/Newsletters/NMICBulletins/NMICBulletins2009/Generic%20bulletin%20NMIC%20v15No1%20web%20with%20refs.pdf
10	Generic Drugs - What are Generic Drugs?	http://www.news-medical.net/health/Generic-Drugs-What-are-Generic-Drugs.aspx&sa=U&ei=EZlPT6aeGsm0hAek5_37Cw&ved=0CEoQFjAL&usg=AFQjCNGQGB3LydlBdSlKOyRYr7zC0uBcxQ
11	GPhA - Generic Pharmaceutical Association	http://www.gphaonline.org/
12	Generic / Brand Drug Name Table	http://www.health.gov.bc.ca/pharmacare/sa/criteria/genericbrandtable.html
13	Generic vs Brand Name Medicines	http://www.patient.co.uk/health/Generic-vs-Brand-Name-Medicines.htm
14	AIDS, Drug Prices and Generic Drugs	http://www.avert.org/generic.htm
15	Canadian Generic Pharmaceutical Association	http://www.canadiangenerics.ca/
16	Generics Are The Same	http://www.genericsarethesame.com/
17	Generic Drugs In Canada: A Policy Paper	http://www.ctac.ca/uploads/Position%20Papers/2007%20EN_PP%20Generic_Drugs_in_Canada_April_2007_FINAL.pdf
18	Benefiting from Generic Drug Competition in Canada: The Way Forward	http://www.competitionbureau.gc.ca/eic/site/cb-bc.nsf/vwapj/GenDrugStudy-Report-081125-fin-e.pdf/$FILE/GenDrugStudy-Report-081125-fin-e.pdf
19	Generic Drugs – The Same Medicine for Less Money	http://www.consumerreports.org/health/resources/pdf/best-buy-drugs/money-saving-guides/english/GenericDrugs-FINAL.pdf
20	Generic Drugs	http://www.medtipster.com/genericdrugs.php
21	The Generic Medicines industry Association of Australia	http://www.gmia.com.au/
22	Australian Prescriber: Frequently asked questions about generic medicines	http://www.australianprescriber.com/magazine/30/2/41/3
23	Pricing of PBS Medicine - Medicare Australia	http://www.medicareaustralia.gov.au/provider/pbs/pharmacists/pricing.jsp
24	Questions and answers on generic medicines	http://www.ema.europa.eu/docs/en_GB/document_library/Medicine_QA/2009/11/WC500012382.pdf

WQA scores of 16 (the second highest WQA score awarded) were given to three websites: (i) Wikipedia’s *Generic drug* page, the highest ranking website by Google search result, (ii) About.com’s page entitled *Generic Drugs: Know the Benefits and Differences of Generic Drugs* and (iii) the FDA’s *Understanding Generic Drugs* (websites numbered 1, 2, and 4 respectively in Table 3). All three websites were situated in the top six websites most observed in the Google SERPs obtained.

The remaining two sites seen in the top six most highly returned websites scored WQA scores of 11 (MedicineNet page, website number 3 in Table 3) and 10 (WHO page, website number 6 in Table 3), indicating that the extent and quality of information in these websites is less than the other four top results, and considerably lower than some of the other websites assessed in this study. This indicates that some websites likely to be seen by searchers contain the relatively poorer or less accurate information on generic medicines.

**Figure 1:**
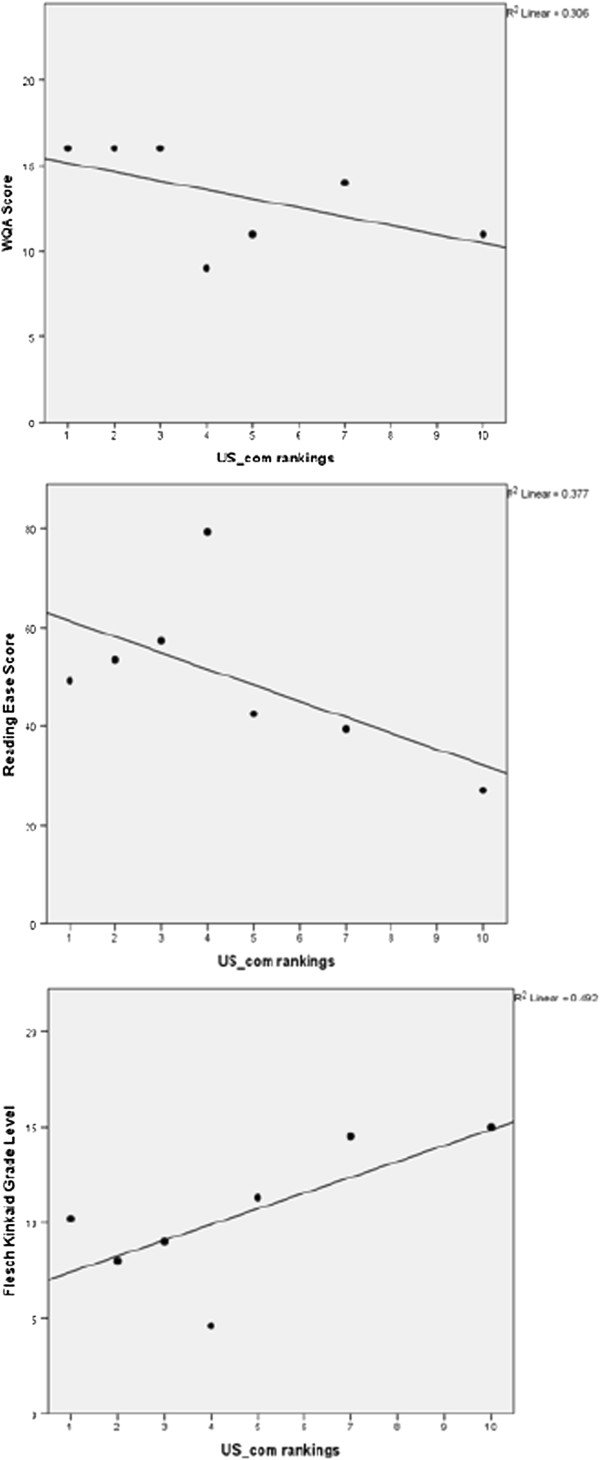
Scatterplots of WQA score, Reading Ease score and Grade level against website ranking on US google.com search.

The association between WQA score and Google search ranking was investigated and a moderate to strong correlation (defined as an absolute value of Spearman’s correlation coefficient > 0.3) was found for searches done in the google.com domain (Figure 1 and Table 4). The most commonly identified websites, i.e., ranked 1, 2 etc., tended to have higher WQA scores. No such correlation was found in the local searches (i.e., google.ie/.co.uk/.ca and .com.au).

**Table 4:** **Correlation between WQA, reading ease score and grade level with ranking using Spearman’s correlation coefficient (r**_**s**_**)**

**Google domain**	**n**	**WQA**		**Flesch reading ease score**		**Flesch Kinkaid grade level**	
		**Spearman’s r**_**s**_	**p-value**	**Spearman’s r**_**s**_	**p-value**	**Spearman’s r**_**s**_	**p-value**
IE / .com	8	−0.49	0.220	−0.33	0.420	0.24	0.570
IE / .ie	10	0.06	0.866	−0.64^*^	0.048^*^	0.58	0.082
UK / .com	8	−0.38	0.352	−0.48	0.233	0.43	0.289
UK / .co.uk	9	−0.51	0.160	−0.58	0.112	0.44	0.232
US / .com	7	−0.67	0.097	−0.64	0.119	0.68	0.094
CA / .com	8	−0.38	0.352	−0.48	0.233	0.43	0.289
CA / .ca	5	−0.70	0.188	0.10	0.873	−0.30	0.624
AU / .com	8	−0.34	0.404	0.29	0.493	−0.38	0.352
AU / .com.au	10	−0.10	0.787	0.00	1.000	0.33	0.359

*Statistically significant at 5% level of significance.

### Accessibility scores

A Flesch Reading Ease score of 60 or greater and a Flesch Kinkaid Grade Level of less than 8 are recommended for general ease of reading.

Three of the websites assessed had a Reading Ease score of greater than or equal to 60: (i) Rx List – Facts about Generic Drugs, (ii) Generic Drugs – The same Medicine for Less Money and (iii) Generic vs Brand Name Medicines (numbered 7, 19, and 13 respectively, in Table 3).

Five of the assessed websites had Grade Level scores of 8 or less: (i) RxList – Facts About Generic Drugs, (ii) Generic Drugs – The Same Medicine for Less Money, (iii) Benefiting from Generic Drug Competition in Canada: The Way Forward, (iv) Generic vs Brand Name Medicines, and (v) Generic Drugs: Know the Benefits and Differences of Generic Drugs – about.com (numbered 7, 19, 18, 13, and 2 respectively, in Table 3).

**Figure 2:**
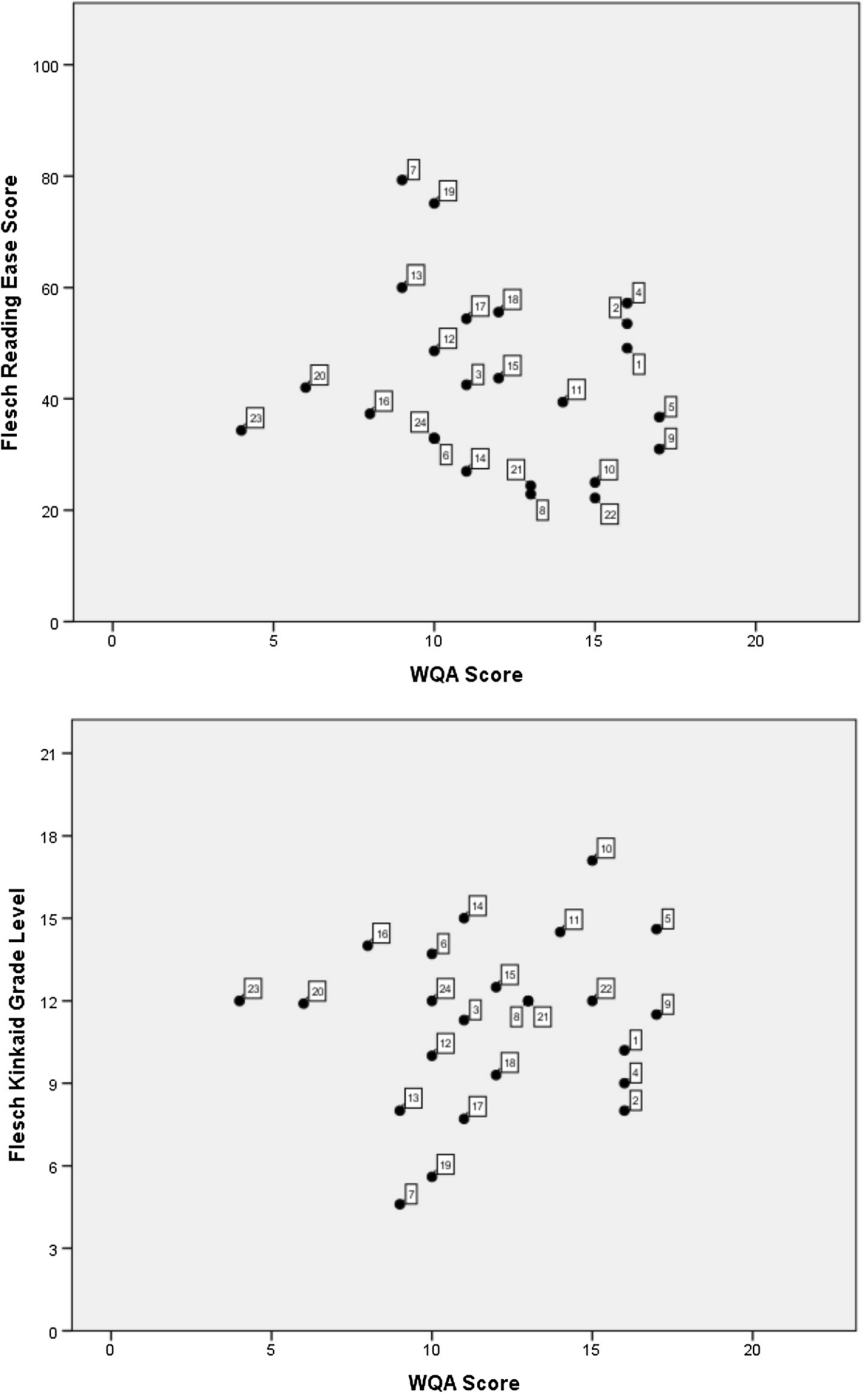
**Scatterplots of readability assessments against WQA score (n = 24 websites).** Note: the numbers refer to website titles in Table 3.

Therefore, as the three websites with the best Reading Ease scores (numbered 7, 19 and 13, respectively, in Table 3) also have appropriate Grade Level scores, it can be determined that those three websites would be the easiest for a member of the public, without a scientific background, to read and understand. However, as those three websites scored relatively low with respect to quality of information they contained - with WQA scores of 9, 10, and 9 respectively (Figure 2) – our study could not assess a site with good readability statistics and containing high quality information. However, analogously to what was demonstrated for WQA scores, Reading Ease scores also demonstrated a relationship with ranking on Google searches. Results from this study indicated that easier to read websites rank higher in Google.com search rankings (Figure 1 and Table 4). Finding statistically significant correlations was limited by the small sample sizes (at most 10 websites in each domain) but a statistically significant correlation was found for the US google.com search (r_s_ = −0.64, p = 0.048). The top ranked sites in all domains also had lower Flesch Kinkaid Grade Level scores (Figure 1 and Table 4).

### Reproducibility

Comparing scores of two independent reviewers (SD and NC) show that, for WQA assessments, almost perfect agreement was seen on average (ICC = 0.94). Similar analysis of readability of the websites using Flesch Reading Ease score and Flesch-Kinkaid Grade Level showed moderate to strong levels of agreement between the two reviewers (ICC value = 0.71 and 0.63, respectively).

Readability scores were assessed by taking a section of text from the website and calculating readability statistics using MS Word. As each rating was independent, different sections of text were likely to be selected from each of the websites assessed. This variation is likely to account for the lower levels of agreement for the reading assessments, compared to the WQA tool. Given the subjectivity of this type of readability assessment, it is reasonable that a moderate consistency was observed throughout the websites assessed.

Overall, the WQA and readabilty scores demonstrate acceptable reproducibilty between two reviewers.

## Discussion

This novel study is the first to assess the websites most likely to be read by a patient searching the Internet for information about generic medicines. More specifically, using the WQA tool developed here, we determined that those websites were all lacking at least some of the information that the authors considered appropriate and relevant for inclusion. However, notably, none of the websites appeared to contain purposefully inaccurate information (as determined by the WQA tool); rather they lacked information that was considered important and that would have gained the website a higher score in our assessments.

Use of Wikipedia as a primary source of information is prevalent and increasing worldwide, [33,34] despite known shortcomings and criticisms, [2,35], including bias and the potential for the information it contains to be corrupted. With use of Wikipedia by clinicians as well as medical students increasing, [35] (and ease of access and ease of understanding being the main reasons cited for its usage amongst medical students), [36] it is reasonable to expect that patients also access this this resource when searching for medical information. Very pertinent to this, is the fact that Wikipedia is available in both English and Simple English – where the Simple version is intended to be more accessible by use of simplified language and limited vocabulary. Indeed, guidelines provided by Wikipedia on writing of Simple information may be useful to those interested in distributing medical information to the general public [37,38].

Recognition of Wikipedia’s prevalence has sparked debate as to whether clinicians should engage in editing Wikipedia to help provide accurate information to patients [39,40]. As the results of this study indicate that patients searching for information on generic medicines in each of the subject countries are likely to find Wikipedia as the first result (Table 2), there appears to be an onus on governments or government-provided healthcare systems to engage with Wikipedia in order to ensure that the information contained therein is impartial as well as accurate. Indeed, given the prevalence of internet –derived information and social networking associated with “web 2.0”, [41] this is an area likely to increase in importance in the future.

Of the other five websites which were most likely to be accessed by a searcher (see Table 2), about.com is a resource website containing articles and other information organized into “channels” on various topics. Freelance writers, referred to as “Guides”, author the articles. About.com differs from Wikipedia in that it does not allow editing by anyone, and it makes use of advertising. The about.com website pages assessed in this study were written by a “patient empowerment guide” without, according to the website, a scientific or medical background. In addition, this website contained frequent, prominent advertising placed in close proximity to the article information, making it possible for searchers to confuse actual information provided with advertising content. The MedicineNet website contained similar levels of advertising to those seen in about.com. However, the author and editor of this page held MD and PhD degrees, respectively, which may endow more credibility. Nonetheless, this website was ranked in the lowest two of the six websites most likely to be seen by searchers, with a WQA score of 11 (see Table 2). The FDA’s page *Understanding Generic Drugs* is a resource likely to be trusted by patients as it is written by the US pharmaceutical regulator. The lack of advertisements also lends the website a more professional, and possibly trustworthy, appearance than some of the other websites assessed during this study. The *netdoctor.co.uk* information on generic medicines, awarded the highest WQA score calculated, was written by a pharmacist. This, as with MedicineNet, may add weight to the website’s content content in a searcher’s opinion. While there are a small number of adverts on this site, they are not as close to the information or as obvious as in other websites discussed above. Finally, the WHO website, while being a reputable source which is likely to be recognised, trusted and possibly even sought out by searchers, unfortunately contains relatively little information useful to the general public when seeking information on generic medicines. Indeed, this was the lowest WQA scoring website of the six most likely to be viewed by searchers. Moreover, it scored low for readability, again reducing the likelihood of it being used by the general public. Interestingly, 7 of the 15 websites that were discarded (i.e., not assessed by WQA) were sales websites. This suggests strongly that a searcher looking for information about generics may encounter a high number of websites selling generic medicines, representing a potential patient safety/public health risk given current concerns with counterfeit medicines being sold online [42]. Only one other website received the highest awarded WQA score (of 17) – a bulletin published by the National Medicines Information Centre at St. James’s hospital in Dublin, Ireland. While the information in this article was of high quality, its readability scores were relatively low. This is probably because the intended audience for this bulletin was healthcare professionals and, thus, the language used in the article would have been appropriate for them. However, while the bulletin provided high quality information, it would have been of little use to a non-scientist. In summary, for websites providing medical information to the general public, quality of information must be allied to language and syntax that matches the reading and comprehension abilities of the intended audience.

In the UK, about 16% of adults are described as “functionally literate”, meaning that they have the literacy levels at or below those expected of an 11-year old [43]. In the Republic of Ireland, the International Adult Literacy Survey revealed that one in four adults have problems with even the simplest of literacy tasks [44], with similar rates being seen in the US [45] and Canada [46]. A key finding of this study was that there is a correlation between good readability statistics and higher ranking on google.com searches (Table 4), indicating that more readable websites are more likely to be found by searchers. However, as the top scoring websites investigated during this study for Reading Ease scored relatively poorly for WQA, we were not able to investigate a website with both good information and good readability. Importantly, the implication is that a searcher looking for information on generic medicines is unlikely to identify a website that is both readable and contains high quality information (as evidenced by a high WQA score). Therefore, there appears to be a gap in knowledge provision that could be filled by a website with high quality information, explaining to the general public specifically what generic medicines are (including dispelling any myths about generic drugs) which is also designed and written to maximize readability. Given the correlations between WQA score and readability statistics and ranking on google.com SERPs evinced by this study, it could reasonably be suggested that (general popularity of sites such as Wikipaedia and the FDA site excepted) such a website would return an enhanced score on a google.com search (across varying IP regions). However, the finding of statistically significant correlations in this study was limited by the small sample sizes, as the study was designed to mimic how a typical searcher would use results of a Google search (i.e., not going beyond the first page of results) [47]. An interesting question arising from this is: who is responsible for provision of such a website? Is it the responsibly of the State to provide good quality, readable medical information to its citizens? Or should it fall to private stakeholders to provide such a service?

## Conclusions

Recommendations from a 2010 report on the proposed model for introduction of generic substitution and reference pricing in Ireland stated that communication of information about generic medicines to the general public would be key for the success of the proposed changed in the Irish healthcare system [48]. Whatever the answer, many patients using the Internet for medical information do not differentiate between high- and low-credibility sources of information when perceiving the quality of the information provided [49] and, therefore, it is clear that medical information websites need to be assessed for quality of information and readability by the intended audience before they are published on the Internet. The WQA tool developed during this evaluation of generic medicine-related site proved effective and relatively easy-to-use in that context, and may, if adapted, be suitable for assessment of other types of medical/healthcare information websites [50].

## Abbreviations

AU: Australia; CA: Canada; EGA: European generic medicines association; FDA: Food and drug administration; ICC: Intraclass correlation coefficient; IE: Ireland; IP: Internet protocol; SERP: Search engine results page; UK: United Kingdom; US: United States; WHO: World health organisation; WQA: Website quality assessment.

## Competing interests

All authors declare that they have no competing interests.

## Authors’ contributions

SD conceived of the idea for the research, designed and conducted the analysis, gathered and interpreted the data and drafted, revised and finalised the manuscript. NC aided in data gathering and interpretation and provided critical review of the manuscript. AH completed statistical analysis of the data, provided critical review of the manuscript and final approval of the version to be published. BS provided critical review of the manuscript and final approval of the version to be published. CD provided critical review of the manuscript and final approval of the version to be published. WC provided critical review of the manuscript and final approval of the version to be published. All authors read and approved the final manuscript.

## Pre-publication history

The pre-publication history for this paper can be accessed here:

http://www.biomedcentral.com/1472-6947/13/115/prepub
